# Coauthorship by patients and other stakeholders with limited knowledge of scientific publishing practices

**DOI:** 10.1186/s12982-021-00105-4

**Published:** 2021-10-21

**Authors:** Steven S. Coughlin

**Affiliations:** 1 Department of Population Health Sciences, Augusta University, 1120 15thStreet, AE-1042, Augusta, GA 30912 USA; 2 Institute of Public and Preventive Health, Augusta University, Augusta, GA USA

There has been extensive discussion about criteria for coauthorship of scientific articles and many scientific journals have adopted or reference the authorship guidelines set by the International Committee of Medical Journal Editors [1]. The ICMJE recommends that authorship be based on the following 4 criteria: (1) substantial contributions to the conception or design of the work; or the acquisition, analysis, or interpretation of data for the work; (2) drafting the work or revising it critically for important, intellectual content; (3) final approval of the version to be published; and (4) agreement to be accountable for all aspects of the work in ensuring that questions related to the accuracy or integrity of any part of the work are appropriately investigated and resolved [1]. Criteria for authorship such as those developed by the ICMJE help to improve author accountability in biomedical research and deter unethical publishing practices such as extending coauthorship to “honorary” coauthors who have not played a substantive role in the research.

In parallel to focused attention on criteria for authorship, there has been increasing interest in extending coauthorship to patients and other stakeholders who may have limited knowledge of scientific publishing practices. This has come about partly because of the success of community-based participatory research (CBPR). CBPR is a collaborative approach to research that equitably involves all partners, including community members affected by the health topic being addressed, organizational representatives, and academic researchers in the research process [2, 3]. This approach includes partnerships between academic and community organizations with the goal of increasing the value of the research product for all partners. CBPR emphasizes shared decision-making, co-learning, reciprocal transfer of expertise between community members and academic partners, and mutual ownership of research products [2]. The CBPR research paradigm represents a fundamental shift in academic researcher’s views of community residents, from patients and research subjects who may benefit from medical advances to essential partners who can energize their communities to develop effective, sustainable interventions to improve health and eliminate health disparities [2]. Community members, organizational representatives, and academic researchers participate in and share control over all phases of the research process including assessment, definition of the problem, selection of research methods, and data collection, analysis, interpretation, and dissemination of findings [2]. In publishing results from CBPR studies, it is common for patients and other community members who have contributed to the research to be invited to be coauthors of study publications.

The extension of coauthorship to patients and other stakeholders who may have limited knowledge of scientific publishing practices has also come about as part of patient-centered outcomes research including studies funded by the Patient-Centered Outcomes Research Institute in the United States. In patient-centered outcomes research, patients who are members of the target population play a key role in identifying research priorities, collecting data, interpreting results, and disseminating findings. Thus, it is not uncommon for patients who have contributed to the research to be invited to coauthor study publications.

The extension of coauthorship to patients and other stakeholders who are unlikely to have experience researching, writing, or publishing scientific manuscripts raises several important issues related to publishing practices and publication ethics. For example, because the nature of research is changing, with increasing participation by nonprofessionals, criteria for authorship may need to be modified to recognize this evolving social dimension of scientific research [4]. Patients and other nontraditional coauthors are unlikely to meet the fourth ICMJE criterion for authorship (agreement to be accountable for all aspects of the work in ensuring that questions related to the accuracy or integrity of any part of the work are appropriately investigated and resolved). In addition, it may not be obvious what contributions should warrant authorship, or who should be responsible for the quality and content of the scientific research findings presented in a publication [5]. A further issue is that patients and other stakeholders with limited knowledge of scientific publishing practices may not be familiar with conflicts of interest that can arise in publishing scientific articles and how to avoid them or manage them.

In order to advance patient-centered outcomes research, CBPR, and other participatory forms of research, educational information is needed in lay language to inform patients and other non-traditional coauthors about scientific publishing practices. Topics that should be discussed include how can patient partners participate in the preparation of manuscripts and dissemination of study findings? What are the roles and responsibilities of coauthors? How do you write an article for publication? Also, how does the peer review process work? By informing patients and other non-traditional coauthors about scientific publishing practices, efforts to make authorship more inclusive can succeed and address scientific and ethical norms regarding criteria for authorship.
